# Ecological interactions affect the bioactivity of medicinal plants

**DOI:** 10.1038/s41598-023-39358-1

**Published:** 2023-07-27

**Authors:** Julia L. Camina, Virginia Usseglio, Victoria Marquez, Carolina Merlo, José S. Dambolena, Julio A. Zygadlo, Lorena Ashworth

**Affiliations:** 1 Instituto Multidisciplinario de Biología Vegetal (IMBIV), Consejo Nacional de Investigaciones Científicas y Técnicas (CONICET), Universidad Nacional de Córdoba (UNC), Córdoba, Argentina; 2 Cátedra de Química Orgánica, Facultad de Ciencias Exactas, Físicas y Naturales (FCEFyN), Universidad Nacional de Córdoba (UNC), Córdoba, Argentina; 3 Cátedra de Química General, Facultad de Ciencias Exactas, Físicas y Naturales (FCEFyN), Universidad Nacional de Córdoba (UNC), Córdoba, Argentina; 4 Facultad de Ciencias Agropecuarias (FCA), Universidad Nacional de Córdoba (UNC), Córdoba, Argentina; 5 Laboratorio Nacional de Análisis y Síntesis Ecológica (LANASE), Universidad Nacional Autónoma de México, Morelia, Mexico

**Keywords:** Ecology, Microbiology

## Abstract

Essential oils produced by medicinal plants possess important bioactive properties (antibacterial, antioxidant) of high value for human society. Pollination and herbivory can modify the chemical defences of plants and therefore they may influence the bioactivity of essential oils. However, the effect of ecological interactions on plant bioactivity has not yet been evaluated. We tested the hypothesis that cross-pollination and simulated herbivory modify the chemical composition of essential oils, improving the bioactive properties of the medicinal plant *Lepechinia floribunda* (Lamiaceae). Through controlled experiments, we showed that essential oils from the outcrossed plant progeny had a higher relative abundance of oxygenated terpenes and it almost doubled the bacteriostatic effect on *Staphylococcus aureus*, compared to inbred progeny (i.e., progeny produced in absence of pollinators). Herbivory affected negatively and positively the production of rare compounds in inbred and outcrossed plants, respectively, but its effects on bioactivity still remain unknown. We show for the first time that by mediating cross-pollination (indirect ecosystem service), pollinators can improve ecosystem services linked to the biological activity of plant’s essential oils. We stress the importance of the qualitative component of pollination (self, cross); an aspect usually neglected in studies of pollination services.

## Introduction

Angiosperms are the core of most terrestrial mutualistic and antagonistic interaction webs^1^. Many angiosperm species establish mutualistic interactions with mycorrhizal fungi and pollinators, which allow them to get specific soil nutrients and sexual reproduction, respectively. In the same way, plants also establish antagonistic interactions with herbivores and detrimental microorganisms, which usually negatively impact plant fitness^1^. Interestingly, plant interactions with organisms as different as fungi, bacteria, animals, and other plants are signaled by secondary metabolites such as terpenes, which are the main components of plant’s essential oils^1–3^. The ecological functions of terpenes include defence against pathogens and herbivores as well as attractants and rewards to beneficial organisms such as mycorrhizal fungi and pollinators^4,5^. Given that terpenes are highly bioactive^6^, essential oils have been widely used by humans in cultural ceremonies and as medicinal, pharmaceutical, agronomic, cosmetic and alimentary resources^3,7–9^. Therefore, essential oils from plants represent benefits that humans obtain from nature (ecosystem services) and their bioactivity may be modeled by ecological interactions. The production and composition of plants’ essential oils are highly variable due to intrinsic and extrinsic factors. Intrinsically, plants can modify the chemical composition and abundance of their essential oils according to their ontogenetic and phenological stages, and to the type of organ (flower, leaf) where it is produced^10^. Moreover, both extrinsic abiotic factors (e.g., light, humidity, nutrient availability)^2,9^, and biotic factors (e.g., mutualistic and antagonistic interactions) can affect the plant chemical profile^2,11–13^.

Herbivory is an antagonistic interaction that is usually considered an ecosystem disservice as it reduces crop yield and favors the transmission of diseases^15^. However, herbivory triggers changes on the chemical composition of plants. Plants produce two types of chemical defences: constitutive defences are those produced continuously, whereas inducible defences are those produced after mechanical and/or herbivore damage^6^. Thus, after herbivory, plants may produce some inducible compound groups that increase their protection against herbivores and pathogens transmitted by herbivores (inducible defences; e.g.,^16,17^). Therefore, some level of herbivory may be beneficial in order to enhance the biological activity of essential oils against other organisms such as insects and microbes (bioactivity). Similarly, pollination quality (i.e., cross- vs. self-pollination) may affect the plant’s chemical constitutive and inducible defences produced after herbivory. The effects of pollination quality on the chemical profile of plants will depend of the plant’s mating system. Indeed, mainly outcrossing and mixed mating plant species have higher probability to undergo inbreeding depression (lower fitness) after self-pollination than selfing plants. It has been shown that inbred plants (originated by self-pollination) experience greater herbivory and increased incidence of diseases than outcrossed plants (originated by cross-pollination)^18,19^. Previous chemical studies support this idea, as plants produced by selfing had reduced expression of constitutive defences and lower ability to up-regulate chemical defences following damage than plants produced by outcrossing^13,20^. Notably, in animal pollinated plants, cross-pollination is mainly carried out by pollinators, while self-pollination may be feasible by both, with (pollinator-mediated selfing) and without (autonomous selfing) pollinators. Thus, cross pollination mediated by pollinators may enhance bioactivity of essential oils. Even more, both interactions, pollination and herbivory, may have synergistic effects on plant bioactivity.

**Figure 1:**
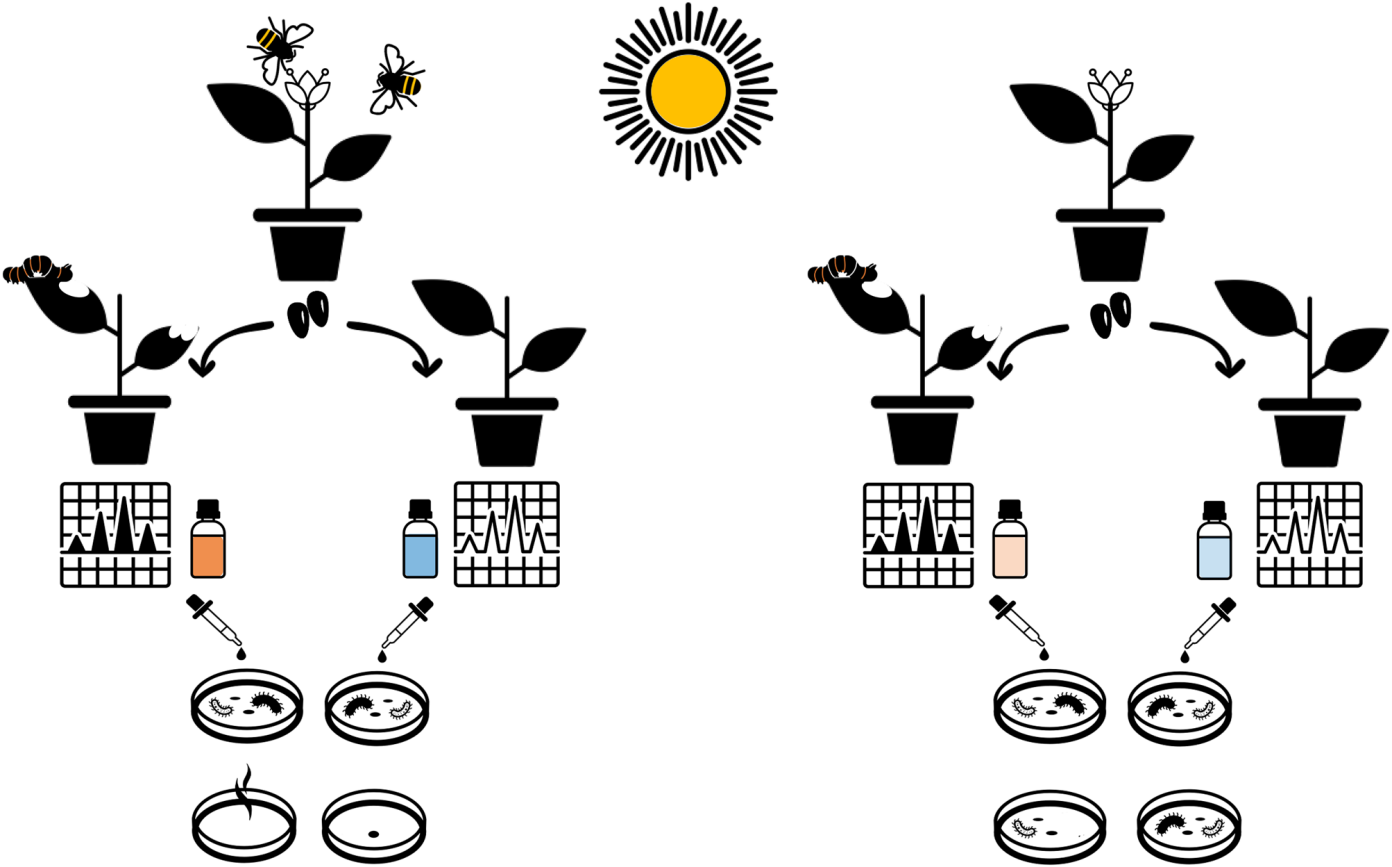
Graphical hypothesis: Cross-pollination and herbivory improve bioactive properties of plants against microorganisms. It is expected that outcrossed plant progeny (left side) have higher bioactive essential oils than inbred plant progeny (absence of pollinators, right side). It is also expected that herbivory increases bioactivity but in higher magnitude in outcrossed plant progeny. Icons used in this figure are from the Noun Project (https://thenounproject.com).

Currently, the enormous gap between chemical studies regarding plant bioactivity and ecological research arenas makes it difficult to know how ecological interactions may affect bioactive properties of plants. Chemical studies testing plant bioactivity do not take ecological interactions into account^7–9^, while studies testing ecological interactions effects analyze plant chemical composition but not bioactivity^21,22^. This knowledge gap is probably a consequence of an originally oversimplified vision of ecosystem services (but see^23,24^). Here, we tested the hypothesis that ecological interactions improve the bioactive properties of the essential oils of *Lepechinia floribunda* (Lamiaceae), a medicinal shrub (Fig. 1). We tested the effect of pollination quality (self and cross) and simulated herbivory on the chemical composition, production, and bioactivity of the essential oils against bacteria species. Essential oils of *L. floribunda* are composed by terpenes with antioxidant, antiviral and antibacterial activities, among others^25–28^. *L. floribunda* is self-compatible and able to autonomously self-pollinate in the absence of pollinators^29^, but its mating system is mostly outcrossing^30^. Thus, it is likely that subjected to self-pollination, *L. floribunda* undergo inbreeding depression. Therefore, we expect that inbred progeny (hereafter inbred plants) will have constitutive chemical defences of lower bioactivity than the outcrossed progeny (hereafter outcrossed plants). We also expect a synergistic effect between pollination quality and simulated herbivory, where inbred plants will have inducible chemical defenses of lower bioactivity than the outcrossed plants.

## Results

### General composition

**Figure 2:**
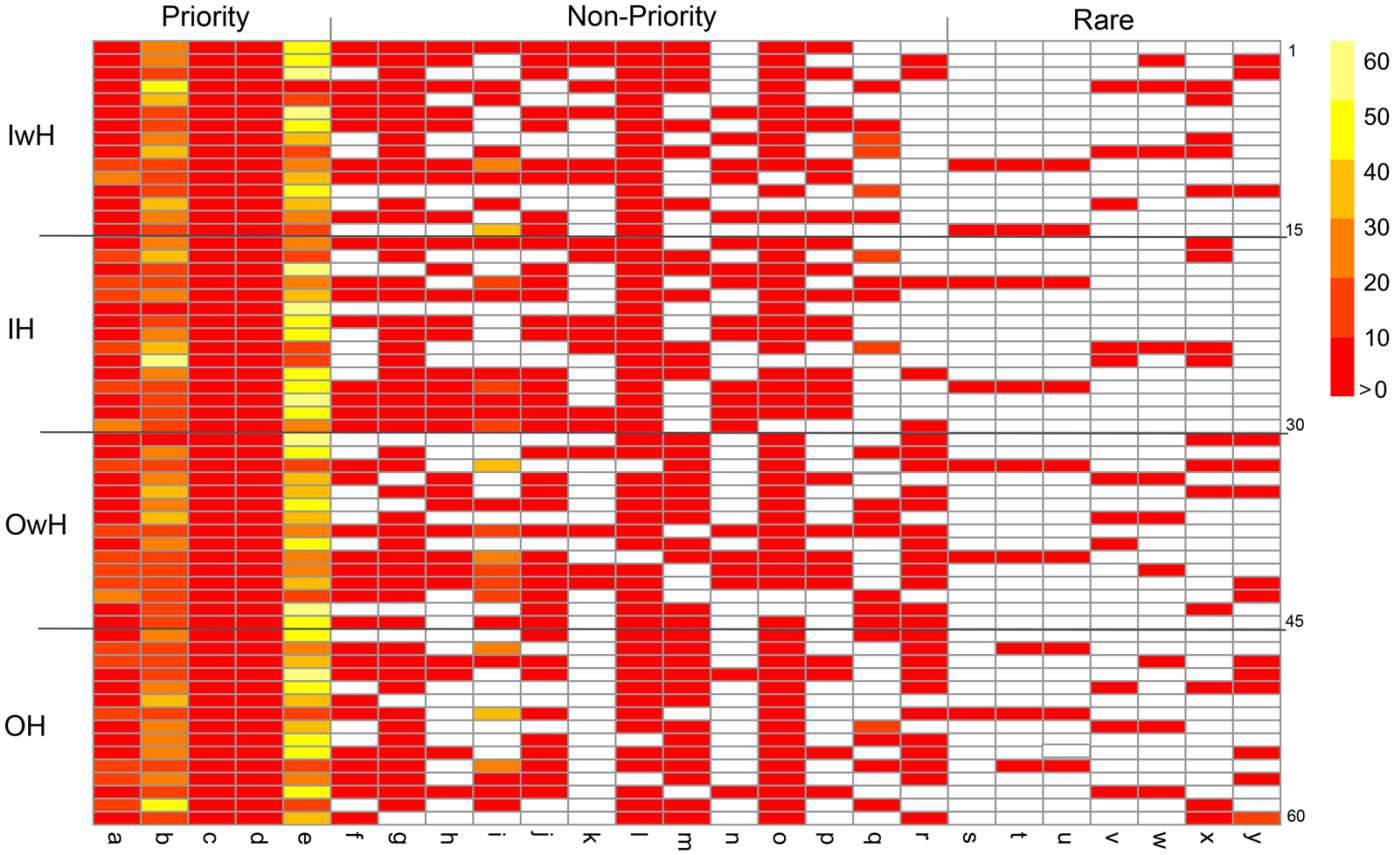
Heatmap showing the relative abundance of the 25 chemical compounds of the essential oil of *Lepechinia floribunda*. Pollination and simulated herbivory treatments, IwH: inbred plants without simulated herbivory, IH: inbred plants with simulated herbivory, OwH: outcrossed plants without simulated herbivory, OH: outcrossed plants with simulated herbivory. Compounds are ordered according to their frequency of occurrence among the tested plants: priority (present in 100% of plants), non-priority (present in less than 100% and more than 30% of plants) and rare compounds (present in less than 30% of plants). Colors shown in the upright scale represent the relative abundances of compounds (i.e., the percentage of × compound in the essential oil of a plant) from more than zero to 60%, and white cells show absence of compounds. Each row represents an individual plant, and each column represents a chemical compound (signaled with letters) a: borneol, b: β caryophyllene, c: aromadendrene, d: α humulene, e: ledol plus unidentified oxygenated sesquiterpene compound, probably a precursor of ledol, f: α terpineol, g: bornyl acetate, h: α gurjunene, i: 1,8 cineol, j: alloaromadendrene, k: α amorphene, l: ledene, m: α fernesene, n: β selinene, o: nerolidol, p: palustrol, q: tau cadinol, r: α bulnesene, s: α pinene, t: camphene, u: β pinene, v: β gurjunene, w: α guaiene, x: γ cadinene, y: α eudesmol. The R software (function pheatmap of the library “pheatmap”, version 4.1.2) was used to create this heatmap.

**Figure 3:**
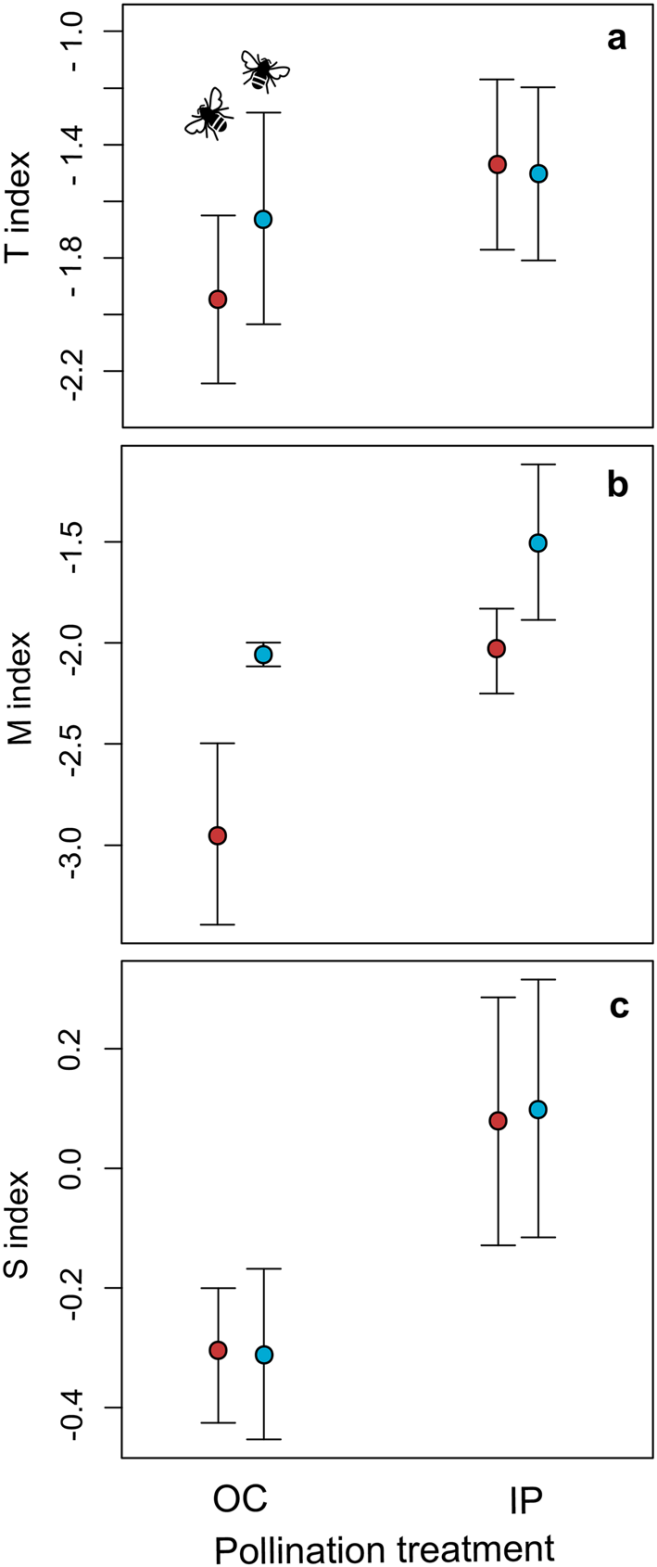
Mean (± SD) relative abundance of the four terpene groups present in the essential oil of *Lepechinia floribunda* (hydrocarbon monoterpenes, oxygenated monoterpenes, hydrocarbon sesquiterpenes and oxygenated sesquiterpenes) expressed as indices. (**a**): T index = ln (relative abundance of monoterpenes/relative abundance of sesquiterpenes). (**b**): M index = ln (relative abundance of hydrocarbon monoterpenes/relative abundance of oxygenated monoterpenes), and **c**: S index = ln (relative abundance of hydrocarbon sesquiterpenes/relative abundance of oxygenated sesquiterpenes). Abbreviations, *OP* outcrossed plants, *IP* inbred plants. Simulated herbivory (red), without simulated herbivory (blue).

In total, 25 different terpenes compounds were found in the essential oil of *L. floribunda* (Fig. 2). The major terpene groups were monoterpenes (oxygenated and hydrocarbon) and sesquiterpenes (oxygenated and hydrocarbon). To test the pollination quality and simulated herbivory effects on the chemical plant’s profile, we conducted a full two factorial experiment with two levels per factor, pollination quality (self and cross) and simulated herbivory (with and without; hereafter treatments). To test changes among treatments in the relative abundance of monoterpenes with respect to sesquiterpenes, we calculated a T index (ln (abundance of monoterpenes/abundance of sesquiterpenes)). The T index was negative for all treatments meaning that the essential oil had higher abundance of sesquiterpenes than monoterpenes in inbred and outcrossed plants and with and without simulated herbivory plants (Fig. 3a). To test changes in the relative abundance of hydrocarbon monoterpenes with respect to oxygenated monoterpenes, we calculated the M index (ln (abundance of hydrocarbon monoterpenes/abundance of oxygenated monoterpenes)). The M index was negative for all treatments meaning that oxygenated monoterpenes were more abundant than hydrocarbon monoterpenes (Fig. 3b). Similarly, to test changes in the relative abundance hydrocarbon sesquiterpenes respect to oxygenated sesquiterpenes, we calculated a S index (ln (relative abundance of hydrocarbon sesquiterpenes/relative abundance of oxygenated sesquiterpenes)). The S index values were both positive and negative (Fig. 3c), indicating that the abundances of oxygenated and hydrocarbon sesquiterpenes were variable among treatments (see detailed results below).

Pollination, simulated herbivory, and the interaction between these factors did not significantly affect the ratio between monoterpenes and sesquiterpenes, T index (Fig. 3a, Table 1). Pollination significantly affected the abundance within the monoterpene group: outcrossed plants had higher relative abundance of oxygenated monoterpenes than inbred plants, as evidenced by the higher negative value of M index (Fig. 3b, Table 1). Pollination also significantly affected the abundance within the sesquiterpene group, with outcrossed plants having higher relative abundance of oxygenated than hydrocarbon sesquiterpenes (S index negative). Inbred plants, in contrast, had similar relative abundance of both kinds of sesquiterpenes (S index close to zero; Fig. 3c, Table 1). These interesting results show that outcrossed plants produced an overall greater abundance of oxygenated than hydrocarbon compounds compared to inbred plants. Simulated herbivory and simulated herbivory*pollination interaction did not significantly affect the relative abundance of oxygenated and hydrocarbon monoterpenes (Fig. 3b), nor the relative abundance of oxygenated and hydrocarbon sesquiterpenes (Fig. 3c; Table 1). Pollination, simulated herbivory and their interaction did not significantly affect the essential oil yield (average oil yield ± SD: 0.17 ± 0.09 ml of essential oil per 100 gr of fresh biomass; Table 1).

**Table 1:** Linear mixed model results of the fixed factors effects and their interaction (Pollination, simulated Herbivory and Pollination* simulated Herbivory) on essential oil yield (ml of essential oil per 100gr of fresh biomass) and composition in *Lepechinia floribunda*.

	Yield	T	M	S
Pollination	*X*^2^ = 0.46; *p* = 0.50	*X*^2^ = 1.74; * p* = 0.19	*X*^2^ = 4.31; * p* = 0.03	*X*^2^ = 4.38; * p* = 0.03
Simulated herbivory	*X*^2^ = 0.004; * p* = 0.85	*X*^2^ = 0.28; * p* = 0.59	*X*^2^ = 2.93; * p* = 0.09	*X*^2^ = 0.01; * p* = 0.91
Pollination* simulated herbivory	*X*^2^ = 1.25; * p* = 0.26	*X*^2^ = 0.09; * p* = 0.77	*X*^2^ = 0.62; * p* = 0.61	*X*^2^ = 0.005; * p* = 0.81
Random factor	30.3%	42.4%	29.26%	42.91%

T index: ln (relative abundance of monoterpenes/relative abundance of sesquiterpenes), M index: ln (relative abundance of hydrocarbon monoterpenes/relative abundance of oxygenated monoterpenes), S index: ln (relative abundance of hydrocarbon sesquiterpenes/ relative abundance of oxygenated sesquiterpenes). Random factor: maternal family. *X*^*2*^ and *p*-values correspond to comparisons between models, the full model (with both fixed factors and their interaction) and the model without the fixed factor of interest. N = 60 plants.

In general, the most abundant monoterpenes were 1,8-cineole, and borneol, followed by α terpineol and bornyl acetate. Among the sesquiterpenes, the most abundant were β-caryophyllene and SL (Ledol + an unidentified oxygenated sesquiterpene, probably an isomer of ledol). The relative abundance of each compound in the essential oil per plant, i.e., the percentage of z compound in the essential oil of a plant, is shown in Fig. 2. Moreover, the list showing the mean (± SD) relative abundances of compounds per treatment, the identity of the compounds (oxygenated and hydrocarbons), and whether they are priority, non-priority and rare compounds is shown in the Supplementary Table S1. It is clearly observed in Fig. 2 and Table S1 that the qualitative composition (presence, absence of compounds) of the essential oil did not differ between pollination treatments, i.e., inbred and outcrossed plants shared 100% of chemical compounds. However, after simulated herbivory, outcrossed plants did not produce α-amorphene (non-priority compound) and inbred plants did not produce α-eudesmol (rare compound, Fig. 2; Supplementary Table S1).

### Quantitative composition

From the 25 compounds found in the essential oil of *L. floribunda*, five of them were priority compounds, being present in all the analyzed plants: borneol, β-caryophyllene, aromadendrene, α-humulene and SL. Thirteen of them were non-priority compounds (present in 30% to <100% of plants), and seven were rare compounds (present in < 30% of plants, Fig. 2; Supplementary Table S1). To test the relative abundance of each priority compound among treatments we first obtained the following ratio per plant: ln (relative abundance of z priority compound/ mean relative abundance of all priority compounds in the plant). Similarly, to test the relative abundance of each non-priority compound per plant we obtained the following ratio: ln (relative abundance of × non-priority compound/mean relative abundance of all non-priority compounds in the plant). The relative abundances of the five priority compounds did not differ between pollination and simulated herbivory treatments (Table 2), but the relative abundance of some non-priority compounds did. Indeed, inbred plants had higher abundance of α-gurjunene, β-selinene, and palustrol than outcrossed plants; meanwhile, in the outcrossed plants there was higher abundance of α-farnesene, nerodinol and α-bulnesene (see Supplementary Table S2 for statistical results of non-priority compounds). After simulated herbivory, outcrossed plants did not produce α-amorphene; i.e., the interaction pollination* simulated herbivory was significant for this compound. Simulated herbivory did not affect the relative abundance of any non-priority compound (Supplementary Table S2). In relation to rare compounds, we found that 66% of inbred and 73% of outcrossed plants (without simulated herbivory) produced one or more rare compounds. After simulated herbivory, the percentage of inbred plants that produced rare compounds decreased (40%) while the percentage of outcrossed plants that produced rare compounds increased (80%; Fig. 2).

**Table 2:** Linear mixed model results of the fixed factors effects and their interaction (Pollination, simulated Herbivory and Pollination* simulated Herbivory) on the relative abundance of priority chemical compounds of essential oils of *Lepechinia floribunda*.

	Borneol	β-caryophyllene	Aroma-dendrene	α-humulene	SL
Pollination	*X*^2^ = 1.26; *p* = 0.26	*X*^2^ = 0.05; *p* = 0.83	*X*^2^ = 3.86; * p* = 0.05	*X*^2^ = 0.77; * p* = 0.38	*X*^2^ = 2.57; * p* = 0.11
Simulated herbivory	*X*^2^ = 0.051; * p* = 0.82	*X*^2^ = 0.20; * p* = 0.65	*X*^2^ = 0.17; * p* = 0.68	*X*^2^ = 0.63; * p* = 0.43	*X*^2^ = 0.14; * p* = 0.70
Pollination * simulated herbivory	*X*^2^ = 0.05; * p* = 0.82	*X*^2^ = 0.29; * p* = 0.59	*X*^2^ = 0.85; * p* = 0.36	*X*^2^ = 0.48; * p* = 0.49	*X*^2^ = 0.001; * p* = 0.90
Random factor	40.68%	32.97%	0.01%	42.83%	48.78%

SL: Ledol plus unidentified oxygenated sesquiterpene compound (probably a precursor of ledol). Random factor: maternal family. *X*^2^ and *p*-values correspond to comparisons between models, the full model (with both fixed factors and their interaction) and the model without the fixed factor of interest. N = 60 plants.

### Bioactivity

The essential oil of *L. floribunda* had bacteriostatic activity against *Staphylococcus aureus* and* Escherichia coli*. The essential oil obtained from the outcrossed plants was significantly more bioactive, having almost twice the bacteriostatic effect than the essential oil obtained from the inbred plants against *S. aureus* (t = 3.13; *p* = 0.020). No differences between inbred and outcrossed plants were observed against *E. coli*. Bactericidal effect was also observed against *S. aureus* and *E. coli*, with both inbred and outcrossed plants showing similar bioactivity. The bactericidal effect against *S. aureus* was reached at 1 ul/ml of essential oil in all the replicates, independently of the pollination treatment (Table 3). Finally, we did not find bacteriostatic nor bactericidal effect of *L. floribunda* essential oil against *Pseudomonas syringae*.

**Table 3:** Bioactivity (bacteriostatic and bactericidal activity) of essential oil of outcrossed (OP) and inbred (IP) *Lepechinia floribunda* plants against two bacterial strains: *Staphylococcus aureus* and *Escherichia coli.*

Pollination treatment	Bacterial strains	MIC (µL/mL) bacteriostatic activity	MBC (µL/mL) bactericidal activity
OP	*Staphylococcus aureus*	0.44 ± 0.12	1 ± 0.00
IP	*Staphylococcus aureus*	0.70 ± 0.27	1 ± 0.00
OP	*Escherichia coli*	2.5 ± 1.73	12 ± 4.62
IP	*Escherichia coli*	2.5 ± 1.73	12 ± 4.62

*MIC* minimal inhibitory concentration, *MBC* minimal bactericidal concentration. Values are mean ± (SD), N = 4 (4 microplates for MIC and 4 agar plates for MBC respectively, per pollination treatment and bacterial species).

## Discussion

In this study, we tested for the first time the influence of ecological interactions on the bioactivity of essential oils of a medicinal plant. We show how pollination quality and herbivory can act as indirect ecosystem services linked to the bioactivity of a medicinal plant (final ecosystem service). We specifically tested the effect of the *qualitative* component of pollination, a neglected aspect in studies of pollination services, where the focus is usually on the *quantitative* component of pollination (i.e., pollinator abundance and floral visitation) on crop yield (e.g.,^31^). Unfortunately, we could not test the effects of simulated herbivory on plant bioactivity, therefore the hypothesis that herbivory may behave as an ecosystem service when the interest lies in plant bioactivity still remains unproven.

Cross-pollination affected both the general and quantitative composition of the essential oils of *Lepechinia floribunda*, whereas simulated herbivory affected the qualitative composition. Essential oils obtained from outcrossed plants had the same qualitative composition (identity of chemical compounds) than inbred plants, but the former had higher relative abundance of oxygenated terpenes. Moreover, the essential oil from the outcrossed plants had almost twice the bacteriostatic effect against *Staphylococus aureus* than the essential oil from inbred plants. Such increased bioactivity of the essential oils from the outcrossed treatment could be explained by the dominance of oxygenated terpenes. Indeed, previous studies have shown greater bioactivity of essential oils when the relative abundance of oxygenated terpenes is greater than that of hydrocarbon ones (e.g.,^9,32,33^). Thus, cross-pollination improved the bioactivity of essential oils by modifying the relative abundances of compounds, but without detectable changes in the qualitative composition.

The abundances of the five priority compounds did not differ between pollination and simulated herbivory treatments, but the abundance of some non-priority compounds differed between pollination treatments. Moreover, a slightly higher percentage of outcrossed than inbred plants produced rare compounds. It is known that the components of the essential oils can act additively, synergically, or antagonistically, and several studies have shown that whole essential oils have a greater antibacterial activity than the priority compounds mixed^7^. Although we did not test how these compounds interact amongst them, our results show that differences in the bioactivity of essential oils between outcrossed and inbred plants were not due to priority compounds, but to the non-priority and/or rare compounds. Indeed, several studies have shown that minor compounds usually have a critical role against bacterial activity (e.g.,^7,14,34^).

As a result of selfing, plants may express inbreeding depression which is a consequence of genetic and epigenetic processes. In this regard, changes observed in the relative abundance of oxygenated terpenes from cross to self-pollination treatments may be the consequence of the expression of deleterious recessive alleles in homozygosity state or the silencing of some genes by DNA methylation in inbred plants^35^. We do not know the specific mechanisms of inbreeding depression involved in our study plant, but our results suggest that the metabolic pathway of oxygenated terpenes would be more negatively affected by inbreeding than the pathway of hydrocarbon terpenes. However, because we used relative abundances of hydrocarbon/ oxygenated terpenes, the opposite is also likely; i.e., the pathway of hydrocarbon terpenes may be upregulated in inbred plants. Future genetic studies testing gene expression will be useful to understand the up/down regulation of specific metabolic pathways as a consequence of inbreeding, see^36^.

Simulated herbivory differentially affected the production of rare compounds. After simulated herbivory, the percentage of outcrossed plants producing rare compounds increased, while the percentage of inbred plants decreased. These results suggest that inbreeding affected gene expression and/or the biosynthesis of inducible defences. As an overall response, we observed upregulation of compounds among outcrossed plants and downregulation among inbred plants. Similar effects of inbreeding on inducible defences against herbivory have been found in previous studies with *Solanum carolinense*^13,20,36^. We were not able to test the effect of simulated herbivory and pollination * simulated herbivory interaction in the bioactivity of essential oils. Yet, if the bioactivity of essential oils of *L. floribunda* is linked to the relative abundance of oxygenated/hydrocarbon terpenes, and simulated herbivory and pollination*simulated herbivory did not affect this trait, then, we would not expect simulated herbivory to have an effect on bioactivity neither. However, simulated herbivory modified the production of rare compounds and, as explained before, minor compounds may also affect bioactivity. Thus, the question whether simulated herbivory affects the bioactivity of *L. floribunda* still remains unanswered.

In this study, we conducted artificial mechanical herbivory to control and standardize the different factors influencing the plant chemical response, see^37,38^. Through simulated herbivory, we kept constant the magnitude of damage to both types of plants. This is a key point to isolate the relative effects of both mutualistic and antagonistic interactions, since inbred plants are usually more damaged by herbivory than outcrossed ones under natural conditions^18,19^. Another important point was to control for the temporal and spatial precision in the application of the damage, as it allows to discard confounding effects produced by the introduction of bacterial, fungal and/or chemical substances present in oral secretions of herbivores^37,38^. Therefore, it is important to highlight that the chemical plant responses that we found in this study may differ from the responses triggered by natural herbivory. We think that manipulative experiments controlling herbivores and the content of their oral secretions may help to understand the isolated effect of mechanical damage and the introduction of microorganisms on plant chemical composition and bioactivity^16,37^.

Among the three strains of bacteria used in this study, essential oils from *L. floribunda* had higher bacteriostatic and bactericidal effect on *Staphylococcus aureus* (Gram +) than on *Escherichia coli* and *Pseudomonas syringae* (both Gram −). The components of essential oils can interfere interbacterial communication and modify the functionality of the bacterial cell membrane through different mechanisms such as increased membrane permeability and fluidity, alteration of ion transport processes, inhibition of respiration, which ultimately conduct to the cell lysis. The differential antibacterial effects we found here are in line with previous findings, where essential oils would be slightly less active against Gram − than Gram + bacteria^7,9^. Such differential effect would be due to differences in the structure of the cellular wall. Gram- bacteria have an extra outer membrane surrounding the cell wall, whereas Gram+ bacteria do not. This extra membrane forms a hydrophilic barrier providing protection against the effects of highly hydrophobic molecules like terpenes. Such morphological differences would explain the differential sensitivity of both types of bacteria to the toxic effects of essential oils.

Here, we have shown for the first time that by mediating cross pollination, pollinators can improve ecosystem services linked to plant bioactivity. We think this framework may be generalized to other ecological interactions and plant species. Our results emphasize the need to broaden our vision about the contributions that society obtain from nature, and to begin quantifying the many other benefits provided by pollinators that go beyond crop production. As an example of such an endeavor, we may begin to test how much pollinators contribute to the sexual reproduction of plants that are managed or harvested from wild populations^39–41^. Additionally, it would be important to disentangle the role of herbivory as an ecosystem service or disservice, depending on the final target benefit obtained from the plants (see^23^ for a broader discussion). Our findings also have direct applied value for production systems focused in plant bioactivity such as crops of medicinal plants and crops of plants used as bioinputs in agroecology. Contrary to what is generally thought, promoting some level of herbivory may increase the bioactive properties of those crops, improving their effects. Finally, we underline the need to better understand the integration of these approaches to incorporate them into production systems.

## Material and methods

### Study species

*Lepechinia floribunda* is a perennial and medicinal shrub distributed between 500 and 3500 m a.s.l. from Bolivia to central-east Argentina. It has white tubular hermaphrodite flowers that are visited by bees, flies and hummingbirds. Moreover, it has some level of autonomous seed production in the absence of pollinators^29,30^. Also, it has some level of herbivory by aphides (*Aphis gossypii* and *Eucarazzia elegans*) and larvae of *Heliothis virescens*, *Heliotis* sp., and some species of Geometridae and Pteropholidae (Lepidoptera)^42^. The essential oils of *L. floribunda* are dominated by terpenes. Although the relative abundance and identity of chemical compounds vary among populations from Bolivia to central Argentina, the main chemical compounds are shared among populations: α-pinene, borneol, canfene, 1,8-cineole, and β-caryophyllene^11,25,26,28^.

### Pollination and simulated herbivory treatments

In November 2013 we selected 21 maternal plants of *Lepechinia floribunda* separated by at least five meters (to decrease the likelihood of selecting seeds from related plants), in a population located in a natural reserve of Chaco serrano subtropical dry forest: “Reserva Hídrica Natural Municipal Los Manantiales” (31° 10′ 21,3′′ S, 64° 20′ 47,5′′ O; Río Ceballos, Córdoba, Argentina). Fifty seeds from each of the selected plants were collected and stored in paper bags at room temperature for eight months. After that, all seeds were scarified with sulphuric acid and put to germinate in Petri dishes under controlled conditions^43^. Thirty seedlings from each maternal plant were sown in pots and grown in a greenhouse under controlled photoperiod (14 h light/10 h dark) with manual irrigation two times a week. After 6 months, when plants started to bloom, we conducted manual cross-pollinations to produce an initial population with null inbreeding; i.e., zero inbreeding coefficient (f = 0). From this progeny we randomly selected 10 individuals per maternal plant to create 16 maternal families (each maternal family had 10 plants) and reserved the remaining 50 individuals (from another 5 maternal plants) to serve as pollen donors. We conducted single-side cross and self-pollinations to maintain the outcrossed progeny and produce the first generation (F1) of inbred progeny, respectively. To increase potential differences among outcrossed and inbred lines, we conducted an additional set of cross and self-pollinations in the following generation (F2). In all cases self- and cross-pollinations were conducted in the same plant. Flowers used for cross-pollinations were emasculated during male phase when the stigma was closed^29^. At the end of this process, we were able to obtain five maternal families as not all the plants reached the reproductive stage in the greenhouse. In September 2016, we performed a greenhouse experiment to measure the effects of inbreeding and simulated herbivory on the production, composition and bioactivity of essential oils. We grew 60 plants in total, six outcrossed plants and six inbred plants per each of the five maternal families. After three months, a treatment of artificial mechanical herbivory was applied to half of the outcrossed and inbred progeny per maternal family. We simulated herbivory (H) with a standard paper hole-punch, making one hole per leaf in all developed leaves of a plant. The remaining progeny was left intact (without simulated herbivory-WH). Plants with and without simulated herbivory were placed in different tables within the greenhouse in order to minimize communication through volatiles amongst them that may trigger induction of defences in plants without simulated herbivory. After 48 h of the simulated herbivory treatment, the aerial part of all plants was harvested and conserved in hermetic plastic bags at – 18 °C until extraction of essential oils. Soil used for all the assays was collected from the natural focal population of *L. floribunda*.

### Extraction, quantification and identification of essential oil compounds

Plant material was defrosted at room temperature (20–25 °C) and weighted in a precision balance (Toledo PB153, precision 2 mg, limit of detection 1mg, Ohaus Corporation, Pine Brook, NJ, U.S.A.). Essential oils were obtained by hydrodistillation of the aerial part of plants (leaves and stems) in a Clevenger-like apparatus for 60 min^44^. The volume of essential oils was quantified with a graduated burette attached to the distiller, and the essential oils yield was estimated as: Yield = (essential oil volume/fresh weight) * 100. Essential oils were stored in Eppendorf at − 18 °C until they were used. The identification and quantification of essential oil compounds were made by gas chromatography-mass and spectrometry analysis using a PerkinElmer Clarus 600 equipped with a DB-5 capillary column (60 m × 0.25mm i.d. and 0.25 μm coating thickness). Analytical conditions were: oven temperature programmed from 60 to 240 °C at 2 °C/min. Helium was the carrier at a constant flow of 0.9 ml/min, and the ionization was 70 eV ion source. The injector was operated in split mode at 250 °C, same as the detector temperature. Essential oil compounds were identified by comparing their retention indices (RI) and the mass spectra with compounds in Wiley and NIST 98 MS Libraries. Besides, some essential oil compounds were confirmed by co-injection with standards (Sigma, USA). We were unable to properly resolve and identify one of the characteristic oxygenated sesquiterpene of the chromatographic profile of *L. floribunda.* This compound appeared in some chromatographic profiles and its peak was overlapped with the peak of ledol, suggesting it to be an isomer of ledol. Thus, for statistical analysis we added the relative abundance of both, the unidentified oxygenated sesquiterpene + ledol and named it SL compound. The relative abundance of a given compound in a plant was obtained as the area under the curve of such compound in the chromatogram/Σ area under the curve of all the compounds in the essential oil of the plant.

### Chemical diversity and composition of essential oils

The chemical diversity and composition of the essential oils were obtained for each plant and compared among pollination and simulated herbivory treatments using two different measures: (1) General composition (through three indices) and (2) Quantitative composition*.*

*General composition*: the relative abundances of the major terpene groups were expressed as indices: T index = ln (relative abundance of monoterpenes/relative abundance of sesquiterpenes), M index = ln (relative abundance of hydrocarbon monoterpenes/relative abundance of oxygenated monoterpenes), and S index = ln (relative abundance of hydrocarbon sesquiterpenes/relative abundance of oxygenated sesquiterpenes). T = 0 indicates similar relative abundance of monoterpenes and sesquiterpenes, positive and negative values indicate higher abundance of monoterpenes (numerator in the formula) and sesquiterpenes (denominator), respectively. The same logical interpretation applies for M and S indexes. For each plant (n = 60) a value of T, M and S were obtained. *Quantitative composition:* the relative abundance of each priority and non-priority compound was tested among treatments.

### Bioactivity

We tested the bioactivity of the essential oil from inbred and outcrossed plants of *L. floribunda* using three pathogenic bacterial strains: *Escherichia coli* ATCC 25922 and *Staphylococcus aureus* ATCC 25923 with clinical importance, and *Pseudomonas syringae* Q KJ569372 with agronomic importance. The bacteriostatic effect of the essential oil was determined by the minimum inhibitory concentration (MIC), which is the lowest concentration of the essential oil capable of inhibiting visible bacterial growth. The bactericidal effect was determined as the minimum bactericidal concentration (MBC), which is the lowest concentration of the essential oil where 99.9 % or more of the initial inoculum was killed. MIC of the essential oil was determined by the broth microdilution method using resazurin as indicator^32^. First, bacteria were incubated overnight at 37 °C in tubes containing Mueller–Hinton broth for *E. coli* and *S. aureus* and at 30 °C in King’s B broth for *P. syringae.* Then, serial tenfold dilutions of each bacterial strain were prepared in Mueller–Hinton 0.15% agar, or King’s B broth 0.15% agar. Later, 170 μl from each dilution in addition with 20 μl dimetilsulfoxide (DMSO), and 10 μl of resazurin (0.01% p/v), were placed in 96-well microplates. The microplates were incubated for 2hs at 37 °C for *E. coli* and S*. aureus*, and for 2hs at 30 °C for *P. syringae* (optimum temperatures). The highest dilution unable to reduce resazurin was considered the appropriate bacterial dilution to work with. Afterwards, four 96-well microplates (two microplates containing *E. coli* and S*. aureus* and two microplates containing *P. syringae*) were prepared with the appropriate dilution of each bacterium as follows: 170 µL of bacterial inoculum with 20 µL of essential oil dilution in order to obtain concentrations of 32,000 ppm, 16,000 ppm, 8000 ppm, 4000 ppm, 3000 ppm, 2000 ppm, 1000 ppm, 500 ppm, 250 ppm, 125 ppm; 170 µL of sterile medium with 20 µL oil diluent (DMSO) (negative control); and 170 µL of bacterial inoculum with 20 µL of DMSO (positive control). The microplates were incubated for 24 h at optimum temperatures. After incubation, 10 µL of resazurin solution was added to all wells and the microplates were incubated again for 2 h. Resazurin is a redox indicator, it is blue when oxidized and pink in its reduced form. Change in color from blue to pink occurs when the surrounding medium is reduced due to dissolved oxygen depletion and acid production consequence of bacterial growth. The MICs were determined visually as the highest dilution with no color change (remained blue^45^). The contents of the wells that remained blue and the controls were then spread on Mueller–Hinton agar plates, and incubated for 48 h at the optimum temperatures to test MBC. The assays to test MIC effects consisted in two microplates (two replicates) per bacterial species, per pollination treatment. Moreover, we replicated the assays in two days (two different weeks), thus the total number of replicates per bacterial species and pollination treatment was N = 4 (two microplates * 2 days). The same number of replicates was used for MBC (two agar plates * 2 days). Unfortunately, we could not test the effect of simulated herbivory on oil bioactivity due to the scarce quantity of essential oil.

Permissions to collect seeds of *Lepechinia floribunda* were obtained from La Secretaria de Ambiente de la Provincia de Córdoba (https://ambiente.cba.gov.ar/). The voucher specimens were deposited at the Museo Botánico de Córdoba (https://museobotanico.unc.edu.ar/protocolo-de-colecciones), Voucher specimens CORD 19852-54 (Ashworth). The experiments were performed under compliance of relevant national and international guidelines and legislations.

### Statistical analysis

Linear mixed models (LMM) were used to test the simultaneous effects of pollination and simulated herbivory on essential oil yield, T, M and S indices, and the relative abundance of priority and non-priority compounds. Pollination and simulated herbivory were fixed factors with two levels each (cross and self-pollination, with and without simulated herbivory, respectively) and maternal family (N = 5) as random factor (*lmer* function of *lme4* package^46^). The statistical significance of fixed factors and their interaction were assessed by *X*^2^ tests, comparing models with and without the factors and interaction of interest. The random factor expresses the percentage of the model variance attributable to variability among maternal families. Because the relative abundance of a given compound (or group of compounds) is not independent of the relative abundance of another compound (or group), we used the Aitchison transformation (ln (x/y))^47^. For example, to test the variability among treatments in the relative abundance of monoterpenes respect to sesquiterpenes we used: T index = ln (relative abundance of monoterpenes/ relative abundance of sesquiterpenes), and to test the relative abundance of a priority compound (e.g., Borneol) we used: ln (relative abundance of Borneol/mean relative abundance of all priority compounds). Rare compounds were absent in many plants, so we could not conduct statistical tests. A t-test was used to compare the Minimum Inhibitory Concentration (MIC, bacteriostatic effect) of essential oils obtained from outcross and self-pollination against *Staphylococcus aureus*. The alpha level (type I error) used in all statistical tests was 0.05, thus the null hypotheses were rejected when *p*-values were ≤ 0.05. All statistical analyses were made with free R software, version 4.1.2^48^.

## Supplementary Information

Supplementary Tables.

## Data Availability

All data generated during the current study are available from the corresponding author upon request.
