# Supplementary material for: Ecological interactions affect the bioactivity of medicinal plants
Source: Sci Rep. 2023 Jul 27;13:12165. doi: 10.1038/s41598-023-39358-1 (PMC10374891; doi:10.1038/s41598-023-39358-1)
Supplement: Supplementary file 1 — Supplementary Tables. [file 41598_2023_39358_MOESM1_ESM.docx]

**Supplementary Information. Camina et al.**

**Supplementary Table S1:** Chemical composition of essential oil of *Lepechinia floribunda.* Treatments: IWH: inbred plants without herbivory, IH: inbred plants with artificial mechanical herbivory, OWH: outcrossed plants without herbivory, and OH outcrossed plants with mechanical herbivory. Values are mean (± SD) of relative abundance. Compounds are listed by elution order from a DB-5 column. LRI: Linear Retention Index. GCMS: gas chromatography-mass spectrometry, GCMS.Co: gas chromatography-mass spectrometry with coinjection of pure compound. SL = relative abundance of ledol plus an unidentified oxygenated sesquiterpene. ^1^priority compounds (present in all tested plants), ^2^non-priority compounds (present in less than the 100% and more than the 30% of tested plants), and ^3^ rare compounds (present in less on the 30% of tested plants). Underlined and non-underlined are oxygenated and hydrocarbon terpenes, respectively.

| **LRI DB-5** | **Chemical compounds** | **IWH** | **IH** | **OWH** | **OH** | **Identification methods** |
| --- | --- | --- | --- | --- | --- | --- |
| 933 | α-Pinene^3^ | 0.57 ± 1.58 | 0.66 ± 1.57 | 0.22 ± 0.57 | 0.11 ± 0.44 | GCMS.Co |
| 951 | Camphene^3^ | 0.98 ± 2.65 | 1.11 ± 2.37 | 0.37 ± 0.97 | 0.27 ± 0.71 | GCMS |
| 979 | β-Pinene^3^ | 0.53 ± 1.39 | 0.56 ± 1.12 | 0.20 ± 0.54 | 0.20 ± 0.54 | GCMS.Co |
| 1039 | 1,8-Cineole^2^ | 7.75 ± 13.97 | 9.57 ± 13.01 | 7.76 ± 10.71 | 6.40 ± 11.59 | GCMS |
| 1181 | Borneol^1^ | 9.99 ± 7.15 | 10.76 ± 5.46 | 10.60 ± 8.24 | 9.03 ± 5.46 | GCMS |
| 1193 | α-Terpineol^2^ | 0.71 ± 0.763 | 0.70 ± 0.68 | 1.03 ± 1.19 | 0.89 ± 0.85 | GCMS |
| 1206 | Bornyl acetate^2^ | 1.52 ± 2.19 | 1.33 ± 0.86 | 0.96 ± 0.94 | 0.89 ± 0 .67 | GCMS |
| 1289 | α-Gurjunene^2^ | 0.46 ± 0.45 | 0.57 ± 0.45 | 0.27 ± 0.35 | 0.20 ± 0.33 | GCMS |
| 1434 | β-Caryophylene^1^ | 22.88 ± 10.12 | 23.98 ± 13.22 | 22.27 ± 8.11 | 22.96 ± 8.09 | GCMS |
| 1451 | β-Gurjunene^3^ | 0.12 ± 0.29 | 0.13 ± 0.28 | 0.11 ± 0.24 | 0.10 ± 0.22 | GCMS |
| 1452 | Aromadendrene^1^ | 2.20 ± 1.29 | 2.27 ± 1.034 | 2.04 ± 1.07 | 1.89 ± 1.14 | GCMS |
| 1458 | α-Guaiene^3^ | 0.11 ± 0.25 | 0.03 ± 0.12 | 0.13 ± 0.27 | 0.14 ± 0.32 | GCMS |
| 1469 | α-Humulene^1^ | 4.01 ± 2.39 | 4.19 ± 2.38 | 3.80 ± 1.43 | 4.28 ± 1.67 | GCMS |
| 1473 | Alloaromadendrene^2^ | 1.49 ± 1.36 | 1.84 ± 1.395 | 1.07 ± 0.94 | 1.08±0,87 | GCMS |
| 1496 | α-Amorphene^2^ | 0.30 ± 0.43 | 0.39 ± 0.50 | 0.14 ± 0.29 | 0.00 ± 0.00 | GCMS |
| 1503 | Ledene^2^ | 1.74 ± 1.26 | 1.68 ± 0.67 | 1.22 ± 0.72 | 1.13 ± 0.89 | GCMS |
| 1509 | α-Farnesene^2^ | 1.23 ± 2.13 | 0.53 ± 0.86 | 1.40 ± 1.13 | 1.88 ± 1.36 | GCMS |
| 1515 | β-Selinene^2^ | 0.26 ± 0.41 | 1.07 ± 1.45 | 0.20 ± 0.42 | 0.22 ± 0.46 | GCMS |
| 1527 | γ-Cadinene^3^ | 0.52 ± 1.32 | 0.41 ± 1.10 | 0.21 ± 0.31 | 0.18 ± 0.34 | GCMS |
| 1571 | Nerolidol^2^ | 1.09 ± 0.89 | 1.00 ± 0.85 | 1.32 ± 1.44 | 1.85 ± 0.68 | GCMS |
| 1589 | Palustrol^2^ | 0.37 ± 0.49 | 0.53 ± 0.61 | 0.15 ± 0.26 | 0.24 ± 0.38 | GCMS |
| 1622 | SL^1^ | 30.55 ± 16.37 | 32.79 ± 18.04 | 38.00 ± 14.18 | 39.73 ± 13.39 | GCMS |
| 1676 | Tau-Cadinol^2^ | 3.21 ± 6.16 | 2.36 ± 5.21 | 3.02 ± 4.22 | 2.57 ± 4.02 | GCMS |
| 1677 | α-Eudesmol^3^ | 0.18 ± 0.45 | 0.00 ± 0.00 | 0.80 ± 1.41 | 1.60 ± 3.43 | GCMS |
| 1684 | α-Bulnesene^2^ | 0.27 ± 0.87 | 0.31 ± 0.81 | 2.53 ± 2.05 | 2.01 ± 1.96 | GCMS |
|  | Hydrocarbon Monoterpenes | 2.20 ± 5.62 | 2.33 ± 5.04 | 0.79 ± 2.05 | 0.58 ± 1.67 |  |
|  | Oxygenated Monoterpenes | 19.96 ± 16.91 | 22.35 ± 17.15 | 20.35 ± 18.57 | 17.21 ± 16.84 |  |
|  | Hydrocarbon Sesquiterpenes | 35.31 ± 15.86 | 37.10 ± 17.36 | 32.86 ± 11.39 | 34.05 ± 9.57 |  |
|  | Oxygenated Sesquiterpenes | 35.67 ± 19.33 | 36.98 ± 18.14 | 45.82 ± 18.19 | 47.98 ± 15.86 |  |

**Supplementary Table S2**. Linear mixed model results of the fixed factors effects (Pollination, Herbivory and Pollination* Herbivory) on the relative abundance of non-priority chemical compounds of essential oils of *Lepechinia floribunda*. Non-priority compounds are those present in less than 100% and more than the 30% of the studied plants. *X*^2^ and p-values correspond to comparisons between models, the full model (with both fixed factors and their interaction) and the model without the fixed factor of interest.

|  | 1-8 Cineole | α-Terpineol | Bornyl acetate | α-Gurjunene | Allo  aromadendrene | α-Amorphene | Ledene | α-Farnesene | β-Selinene | Nerolidol | Palustrol | Tau-Cadinol | α-Bulnesene |
| --- | --- | --- | --- | --- | --- | --- | --- | --- | --- | --- | --- | --- | --- |
|  |  |  |  |  |  |  |  |  |  |  |  |  |  |
| Pollination | *X*^2^=1.01; p=0.31 | *X*^2^=0.02; p=0.88 | *X*^2^=1.69; p=0.19 | *X*^2^=13.54; p=0.0002 | *X*^2^=1.00; p=0.31 | *X*^2^=11.43; p=0.0007 | *X*^2^=3.80; p=0.51 | *X*^2^=23.92; p=0.000001 | *X*^2^=10.22; p=0.001 | *X*^2^=7.72; p=0.005 | *X*^2^=7.47; p=0.006 | *X*^2^=0.29; p=0.59 | *X*^2^=46.73; p=0.0000001 |
| Herbivory | *X*^2^=0.47; p=0.83 | *X*^2^=0.05; p=0.82 | *X*^2^=0.59; p=0.44 | *X*^2^=0.07; p=0.79 | *X*^2^=0.06; p=0.81 | *X*^2^=1.48; p=0.22 | *X*^2^=0.09; p=0.76 | *X*^2^=0.09; p=0.75 | *X*^2^=0.92; p=0.34 | *X*^2^=0.67; p=0.41 | *X*^2^=0.0002; p=0.99 | *X*^2^=0.08; p=0.77 | *X*^2^=0.160; p=0.69 |
| Pollination*Herbivory | *X*^2^=0.41; p=0.52 | *X*^2^=1.93; p=0.16 | *X*^2^=1.45; p=0.23 | *X*^2^=1.05; p=0.30 | *X*^2^=0.02; p=0.62 | *X*^2^=8.23; p=0.004 | *X*^2^=0.08; p=0.78 | *X*^2^=0.96; p=0.33 | *X*^2^=0.87; p=0.35 | *X*^2^=1.26; p=0.23 | *X*^2^=0.21; p=0.64 | *X*^2^=0.017; p=0.89 | *X*^2^=0.14; p=0.71 |
| Maternal family (random factor) | 11% | 35% | 18.83% | 25.76% | 30.94% | 0.001% | 5.50% | 39.61% | 14.27% | 7.78% | 17.54% | 24.26% | 35.18% |
